# What is the relationship between serum uric acid level and insulin resistance?: A case-control study

**DOI:** 10.1097/MD.0000000000036732

**Published:** 2023-12-29

**Authors:** Ayça Asma Sakalli, H. Seda Küçükerdem, Olgu Aygün

**Affiliations:** a Department of Family Medicine, Balikesir Atatürk City Hospital, Gaziosmanpaşa, Turkey; b Department of Family Medicine, Health Science University, Izmir Bozyaka Training and Education Hospital, Izmir, Turkey.

**Keywords:** cholesterol, HDL, insulin resistance, triglycerides, uric acid

## Abstract

Diabetes, arises from either an absolute or relative insufficiency of insulin or insulin resistance of peripheral tissues. For assessing long-term blood glucose concentration and insulin resistance, the utilization of glycosylated hemoglobin (HbA1c) and the Homeostatic Model Assessment of Insulin Resistance (HOMA-IR) is widespread. Insulin resistance can lead to hyperuricemia by reducing the kidney ability to excrete urate, thus increasing sodium reabsorption. The aim of this study was to investigate the possible relationship between serum uric acid levels and insulin resistance. This was a retrospective case-control study. A total of 2530 applications in 2-year time were included in the study. Patient, known hypertension status, fasting plasma glucose, insulin, uric acid, HDL, low-density lipoprotein (LDL), triglyceride/Tg, HbA1c laboratory values and Tg/HDL ratio were examined. A statistically significant difference existed in the median uric acid values between the insulin-resistant and insulin-sensitive groups (*P* < .001). Additionally, a weak positive statistical correlation was identified between uric acid and HOMA-IR values (*R* = 0.299; *P* < .001) and uric acid and Tg/HDL values (*R* = 0.357; *P* < .001). This study concludes that there is a positive correlation between serum uric acid levels and insulin resistance.

## 1. Introduction

Diabetes, a multisystemic disorder, is caused by absolute or relative insulin deficiency or peripheral tissue resistance to insulin. The prevalence of insulin resistance in adults in Europe is 15%^.[1]^

Glycosilated hemoglobin (HbA1c) and homeostatic assessment of insulin resistance (HOMA-IR) are supportive tests for assessing diabetes and insulin resistance. While HbA1c reflects the 3-month average blood glucose concentration, HOMA-IR is a calculation model used to evaluate insulin resistance. Recently, various parameters such as triglyceride/glucose (TyG) index, triglyceride/high-density lipoprotein (Tg/HDL) ratio, body mass index, waist circumference, and visceral adiposity index have shown promising results as markers for the assessment of insulin resistance.^[2]^

Insulin resistance reduces renal excretion of uric acid on the proximal tubular of the kidney leading to hyperuricemia. In rats, the administration of insulin decreased urinary urate excretion, with concurrent increased expression of a major urate reabsorption transporter (URAT1) and decreased expression of a major urate secretory transporter (ABCG2).^[3]^ GLUT9, which transports glucose and fructose, is expressed in the proximal renal tubular cell and is a high-capacity urate transporter. Insulin activates GLUT9a, the specialized basolateral outflow pathway for urate reabsorption in the proximal tubule.^[4]^

Uric acid, a byproduct of purine metabolism, has been recognized as a mediator in pathological processes, including oxidative stress, inflammation, and endothelial dysfunction^.[5]^

Uric acid significant role in gout and the formation of kidney stones is widely acknowledged. Apart from crystalline arthropathy and urolithiasis, there is a large body of literature suggesting a possible etiologic role of elevated uric acid levels in the pathogenesis of cardiovascular risk factors and hypertension, atrial fibrillation, chronic kidney disease, heart failure, coronary artery disease and cardiovascular death.^[6,7]^

The direct relationship between elevated uric acid levels and the development of diabetes remains a subject of ongoing debate. There is strong evidence suggesting that high serum uric acid levels may contribute to pancreatic β-cell damage.^[8]^ Elevated UA levels cause β-cell injury via the NF-kappa B–iNOS–NO signaling axis.^[9]^ Uric acid may phosphorylate insulin receptor substrate 1 and Akt, resulting in insulin signaling suppression. By raising the activity of nicotinamide adenine dinucleotide phosphate (NADPH) oxidase and xanthine oxidase, hyperuricemia may also enhance the formation of reactive oxygen species (ROS). By lowering NO bioavailability, ROS can decrease NO-cGMP-dependent GLUT4 translocation and glucose absorption.^[10]^ ENPP1 (ectonucleotide pyrophosphatase/phosphodiesterase 1) is a gene that can impede insulin receptor function and is overexpressed in insulin-resistant individuals. Through the recruitment of ENPP1, uric acid directly interacts with the insulin signaling pathway in endothelial cells.^[11]^

Various programs are being implemented worldwide to prevent diabetes. The primary goal of these programs is to identify individuals at risk of diabetes and prevent its development through lifestyle changes and various medical interventions. A better understanding of the relationship between hyperuricemia and insulin resistance could contribute to the early detection of insulin resistance, a precursor to diabetes, in individuals with detected hyperuricemia.^[12]^ This study aimed to investigate the possible relationship between uric acid levels and insulin resistance.

## 2. Materials and methods

### 2.1. Study population & data collection

The study was structured as a retrospective case-control study. Records of patients, aged 18 and above, who visited a family medicine outpatient clinic at a training and research hospital between 2021 and 2023 in Izmir for various reasons. In this study, the principles of the Declaration of Helsinki were followed and approval was obtained from the ethics committee of Izmir Bozyaka Training and Research Hospital on 22.03.2023 with decision number 2023/27.

**Figure 1.**
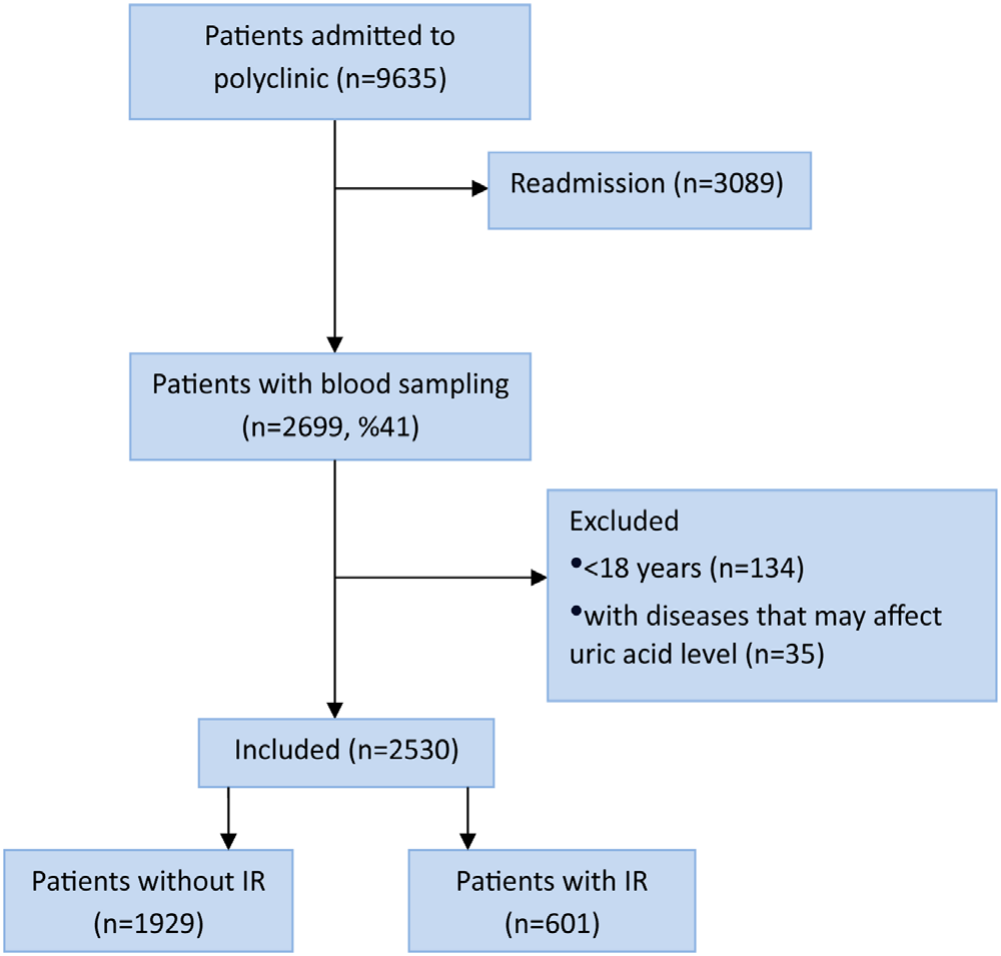
Material-method diagram.

From a total of 9635 outpatient records, 3089 duplicate applications were eliminated from the study. Patients without laboratory results in their records, those under 18 years old, and individuals with conditions that might impact uric acid levels (such as bleeding diathesis, gout, purine metabolism disorder, polycythemia, renal failure, polycystic kidney disease, psoriasis, chronic liver disease, malignancy, congestive heart failure, and those taking medications influencing plasma uric acid levels) were excluded from the study. 2530 people were included in the study (Fig. 1).

### 2.2. Laboratory

Demographics of patients such as age, gender, known hypertension, chronic disease status, and laboratory values of fasting plasma glucose, insulin, uric acid, HDL, low-density lipoprotein (LDL), triglycerides, and HbA1C were recorded. The Homeostatic Model Assessment of Insulin Resistance (HOMA-IR) was computed using the insulin sensitivity index (fasting plasma glucose × insulin/ 405). Patients were categorized into 2 groups according to their IR status: insulin resistant and insulin sensitive. Patients with a HOMA-IR score ≥ 2.5 were classified as having insulin resistance. Patients with a HOMA-IR score < 2.5 were defined as insulin sensitive.^[13,14]^ The established upper limit for uric acid in our hospital laboratory is 7.2 mg/dL. Based on this threshold, patients were categorized as having low or high uric acid values.

### 2.3. Statistical method

Data were analyzed with IBM SPSS V23. Compliance with normal distribution was examined by Kolmogorov-Smirnov Test. Pearson Chi-Square Test was used to analyze categorical variables according to groups. Mann–Whitney *U* test was used to compare the parameters that did not conform to normal distribution according to the groups. Spearman rho Correlation Coefficient was used to examine the relationship between parameters that did not fit the normal distribution. ROC Analysis was used to determine the cutoff value for the uric acid parameter in determining the presence of insulin resistance. Analysis results were presented as frequency (percentage) for categorical variables, mean ± standard deviation, and median (minimum-maximum) for quantitative variables. The significance level was taken as *P* < .050.

## 3. Results

In the group of 2530 participants who met the inclusion criteria, the mean age was 54.07 (18–94) years. Of the participants, 754 (29%) were male and 1776 (71%) were female. A statistically significant difference was found between the gender distributions of the participants according to the groups (*P* < .001). The proportion of men was 27.7% in those without insulin resistance and 36.4% in those with insulin resistance. The proportion of women was 72.3% in those without insulin resistance and 63.6% in those with insulin resistance. While the rate of hypertension in those without insulin resistance was 25.8%, this rate was 37.7% in those with insulin resistance (*P* < .001).

As seen in Table 1, a statistically significant difference was found between the median values of uric acid according to the groups (*P* < .001). While the median value of uric acid was 4.6 in those without insulin resistance, the median value was 5.5 in those with insulin resistance. A statistically significant difference was found between the median values of total cholesterol according to the groups (*P* = .010). While the median value of total cholesterol was 215 in those without insulin resistance, it was 207 in those with insulin resistance. A statistically significant difference was found between the median values of triglycerides according to the groups (*P* < .001). While the median value of triglycerides was 107 in those without insulin resistance, the median value was 149.5 in those with insulin resistance. A statistically significant difference was found between the median values of Tg/HDL according to the groups (*P* < .001). While the median value of Tg/HDL was 1.82 in those without insulin resistance, the median value was 3.05 in those with insulin resistance.

**Table 1:** Comparison of the ages and laboratory values of the participants according to the groups (n = 2530).

		Insulin-sensitive (N = 1929)	Insulin-resistant (N = 601)	*P* *
		Median (min-max)	Median (min-max)	
Age (yr)		54 (18–94)	60 (18–94)	**<.001**
History of HT	Yes	498 (25.8)	227 (37.7)	**<.001**
	No	1431 (74.2)	374 (62.3)	
History of DM	Yes	280 (14.5)	220 (36.6)	**<.001**
	No	1649 (85.5)	381 (63.4)	
Fasting plasma glucose (mg/dL)		86 (25.8–441)	98 (65–356.9)	**<.001**
Uric acid (mg/dL)		4.6 (0.6–21)	5.5 (2.21–11.32)	**<.001**
Total cholesterol (mg/dL)		215 (1–468)	207 (1–372)	**.010**
Triglycerides (mg/dL)		107 (17–822)	149.5 (16–897)	**<.001**
LDL (mg/dL)		131 (9–345)	124 (26–331)	**.004**
HDL (mg/dL)		58 (4–142)	49 (3–116)	**<.001**
Insulin (mU/L)		6.16 (0.64–25.45)	14.43 (4.4–78.52)	**<.001**
HbA1c (%)		5.7 (4–11.7)	5.95 (4.2–28)	**<.001**
HOMA-IR		1.18 (0–2.5)	3.5 (2.51–57.05)	**<.001**
Tg/Hdl		1.82 (0–21.92)	3.05 (0.28–76.33)	**<.001**

Bold values are statistically significant.

LDL = low-density lipoprotein.

* Mann–Whitney *U* test

**Figure 2.**
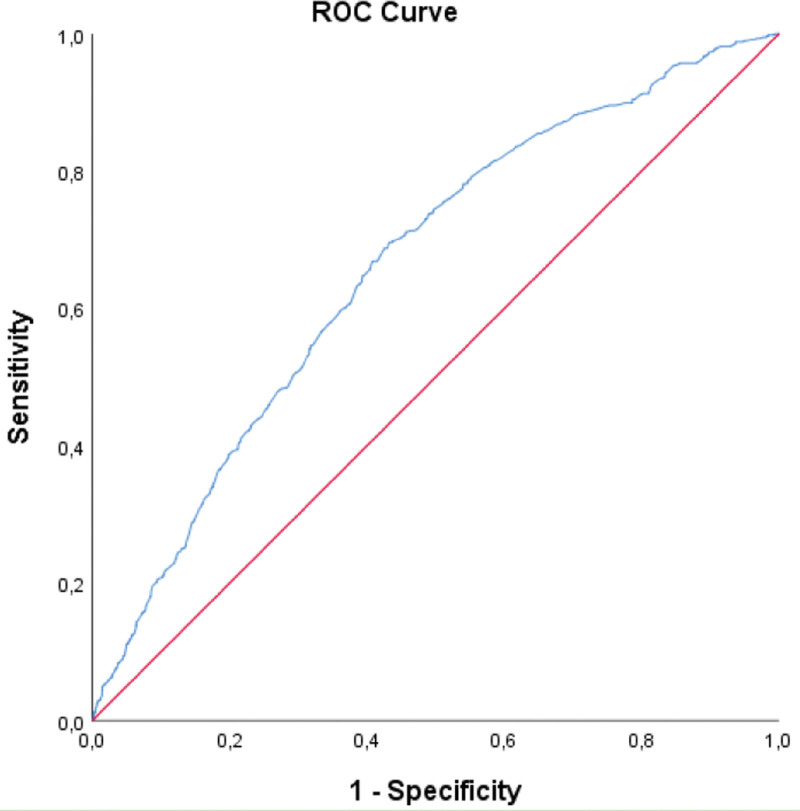
ROC curve for the uric acid parameter.

A significant cutoff value was found for the uric acid parameter in determining the presence of insulin resistance (AUC = 0.660; *P* < .001). The cutoff value for uric acid was found to be ≥ 4.86. Sensitivity was 69.45%, specificity was 56.84%, PPV was 36.67% and NPV was 83.80% (Table 2). ROC curve for uric acid parameter is shown in Figure 2.

**Table 2:** Determination of the cutoff value for the uric acid parameter in determining the presence of insulin resistance.

	cutoff	AUC (%95 CI)	*P*	Sensitivity (%)	Specificity (%)	PPV (%)	NPV (%)
Uric acid (mg/dL)	≥4.86	0.660 (0.635–0.685)	**<.001**	69.45%	56.84%	36.67%	83.80%

Table 3 shows the relationship between uric acid and age and laboratory parameters in all patients. There is a statistically significant positive and weak correlation between uric acid and fasting plasma glucose values (*R* = 0.233; *P* < .001). There is a statistically significant positive weak correlation between uric acid and Tg values (*R* = 0.304; *P* < .001). A statistically significant weak negative correlation exists between uric acid and HDL values (r = −0.309; *P* < .001). There is a statistically significant positive weak correlation between uric acid and insulin values (*R* = 0.29; *P* < .001). There is a statistically significant positive weak correlation between uric acid and HbA1C values (*R* = 0.248; *P* < .001). A statistically significant positive weak correlation exists between uric acid and HOMA-IR values (*R* = 0.299; *P* < .001). A statistically significant positive weak correlation exists between uric acid and Tg/HDL values (*R* = 0.357; *P* < .001).

**Table 3:** Examination of the relationship between uric acid level and age and laboratory parameters in all patients (n = 2530).

		Uric acid
Age (yr)	r	0.268
	p	**<0.001**
Fasting plasma glucose (mg/dL)	r	0.233
	p	**<0.001**
	p	**<0.001**
	p	**<0.001**
Total cholesterol (mg/dL)	r	0.001
	p	0.967
Triglycerides (mg/dL)	r	0.304
	p	**<0.001**
LDL (mg/dL)	r	0.002
	p	0.924
HDL (mg/dL)	r	−0.309
	p	**<0.001**
Insulin (mU/L)	r	0.290
	p	**<0.001**
	p	0.913
HbA1c (%)	r	0.248
	p	**<0.001**
HOMA-IR	r	0.299
	p	**<0.001**
Tg/HDL	r	0.357
	p	**<0.001**

LDL = low-density lipoprotein, r = Spearman rho Correlation Coefficient.

Insulin resistance is related to uric acid (*P* < .001). The rate of low uric acid was 94.5% and the rate of high uric acid was 5.5% in those without insulin resistance. In those with insulin resistance, the rate of low uric acid was 88.2% and the rate of high uric acid was 11.8% (Table 4).

**Table 4:** Comparison of uric acid categories according to insulin resistance.

Uric acid category	Insulin-sensitive (%)	Insulin-resistant (%)	Test statistic	*P* *
Low (≤ 7.2 mg/dL)	94.5	88.2	25.265	**<.001**
High (>7.2 mg/dL)	5.5	11.8		

* Pearson Ki-square test.

Binary Logistic Regression Analysis was used to analyze the risk factors affecting uric acid elevation and the model was analyzed as univariate and multiple. When the model was analyzed as univariate; increasing age value increases the risk of uric acid elevation 1023 times (*P* < .001). Increasing fasting plasma glucose (FPG) value increases the risk of uric acid elevation 1.014 times (*P* < .001). Increasing Triglycerides value increases the risk of elevated uric acid 1.008-fold (*P* < .001). Increasing HDL value had a protective effect on uric acid elevation (OR = 0.962; *P* < .001). Increasing HbA1c value increases the risk of uric acid elevation 1.394-fold (*P* < .001). Increasing HOMA-IR value increases the risk of uric acid elevation 1.369-fold (*P* < .001). Increasing Tg/Hdl value increases the risk of elevated uric acid 1.362-fold (*P* < .001) (Table 5).

**Table 5:** The value obtained from the ROC Analysis and the value affecting the high uric acid value analyzing risk factors.

	Univariate		Multiple	
	OR (%95 CI)	*P*	OR (%95 CI)	*P*
Age (years)	1023 (1018–1029)	**<.001**	1006 (0.999–1013)	.098
Fasting plasma glucose (mg/dL)	1014 (1009–1018)	**<.001**	0.993 (0.987–1)	.051
Total cholesterol (mg/dL)	1 (0.998–1001)	.729	0.998 (0.99–1007)	.710
Triglycerides (mg/dL)	1008 (1006–1009)	**<.001**	1004 (1001–1006)	**.002**
LDL (mg/dL)	1 (0.998–1002)	.806	1002 (0.993–1011)	.659
HDL (mg/dL)	0.962 (0.956–0.968)	**<.001**	0.998 (0.986–1.01)	.711
HbA1c (%)	1394 (1232–1577)	**<.001**	1011 (0.877–1166)	.876
HOMA-IR	1369 (1284–1458)	**<.001**	1295 (1191–1407)	**<.001**
Tg/Hdl	1362 (1287–1441)	**<.001**	1456 (1227–1728)	**<.001**

Cox & Snell R^2^ = %28.3; Nagelkerke R^2^ = %37.7.

LDL = low-density lipoprotein.

When the model was analyzed as multiple; Increasing triglycerides value increases the risk of uric acid elevation 1.004 times (*P* = .002). Increasing HDL value had a protective effect on uric acid elevation (OR = 0.962; *P* < .001). Increasing HbA1c value increases the risk of uric acid elevation 1.394-fold (*P* < .001). Increasing HOMA-IR value increases the risk of elevated uric acid 1.369-fold (*P* < .001). Increasing Tg/Hdl value increases the risk of elevated uric acid 1.456-fold (*P* < .001) (Table 5).

## 4. Discussion

In this study, we evaluated the relationship between serum uric acid levels and insulin resistance in 2530 patients retrospectively.

There are studies supporting that elevated uric acid concentrations independently increase the risk of diabetes, regardless of other known risk factors.^[15]^ In a study conducted with 5821 patients, elevated uric acid levels exhibited a positive correlation with both adipose tissue insulin resistance index and HOMA-IR (*P* < .001).^[16]^ In a laboratory study with mice, insulin resistance and impaired glucose tolerance were observed in hyperuricemic mice, providing the first evidence that high uric acid levels directly induce insulin resistance in vivo and in vitro.^[17]^ Another study confirmed a unidirectional relationship from uric acid to hepatic insulin resistance (IR) and peripheral IR, providing evidence that uric acid is likely a causal factor of IR.^[18]^

According to our findings, there is a statistically significant relationship between insulin resistance and serum uric acid levels. In our study, we observed a statistically significant difference in the median uric acid values between the group with insulin resistance and the group without insulin resistance (*P* < .001). While the median uric acid value was 4.6 for individuals without insulin resistance, it was 5.5 for those with insulin resistance. In the study by Niu et al, the median value of uric acid was 351.50 umol/L (3.97 mg/dL) in the group with a low HOMA-IR score and 415 umol/L (469 mg/dL) in the group with high HOMA-IR score.^[19]^ Similar to our study, the referenced research discovered a noteworthy correlation between uric acid and insulin resistance.

In our study, the established cutoff value for the uric acid parameter to identify the presence of insulin resistance was ≥ 4.86 (AUC = 0.660; *P* < .001). Martínez-Sánchez et al noted that the optimal sensitivity and specificity for identifying individuals with insulin resistance were observed at a value of 5.5 mg/dL, suggesting that serum uric acid levels could serve as a valuable biochemical marker for insulin resistance in primary prevention.^[20]^

Serum uric acid levels are associated with endothelial dysfunction, oxidative stress, and inflammation. In a meta-analysis, uric acid levels were associated with an increased risk of all-cause mortality and stroke in Type 2 diabetes mellitus patients (T2DM).^[21]^ In a cross-sectional study involving 354 participants, it was observed that serum uric acid concentration exhibited a significant correlation with both insulin resistance and impaired insulin secretion.^[20]^

Furthermore, some studies present conflicting results, indicating a lack of significant association between serum uric acid levels and the development of insulin resistance and diabetes mellitus. For instance, Li et al conducted a prospective cohort study involving 4412 non-diabetic patients and found no evidence linking uric acid concentration to an increased risk of T2DM.^[22]^ Hu et al reported an association between higher plasma uric acid levels and an elevated risk of insulin resistance, further noting a gender-based disparity in this relationship. Nevertheless, their study does not offer evidence to substantiate the idea of a causal role of plasma uric acid in contributing to insulin resistance in patients with newly diagnosed T2DM.^[23]^

In our study, there was a weak positive statistically significant correlation between uric acid and FPG values (*R* = 0.233; *P* < .001). In a prospective study, it was found that the FPG levels of people with increased serum uric acid levels were also significantly increased.^[24]^ In a study conducted in northern Italy, increased uric acid was associated with an increased risk of developing impaired fasting glucose ([relative risk (RR) = 1.26, confidence interval (CI) 1.06–1.5, *P* = .01]).^[25]^

Several studies indicates that fasting insulin levels alone might accurately reflect insulin resistance. In a retrospective study, uric acid was positively correlated with hyperinsulinemia and insulin resistance in patients with prediabetes. This result was identified as a potential risk factor for these conditions.^[26]^ Han et al demonstrated a positive correlation between elevated serum uric acid levels and the risk of hyperinsulinemia, as well as a positive correlation between serum uric acid and the risk of insulin resistance (IR) in all individuals except postmenopausal subjects.^[27]^ In our study, we found a weak positive statistical correlation between uric acid and insulin levels (*R* = 0.29; *P* < .001). A mendelian randomization study indicated that hyperinsulinemia leads to hyperuricemia, but not vice versa. While addressing insulin resistance might lower the risk of elevated uric acid levels and gout, decreasing uric acid levels does not appear to mitigate insulin resistance or its cardiovascular implications.^[12]^ In contrast to this study, a meta-analysis of randomized controlled clinical trials revealed that interventions aimed at reducing uric acid levels resulted in decreased insulin resistance and lowered blood pressure. However, these interventions did not yield a significant effect on HOMA-β and serum lipid levels.^[28]^

There is a high degree of variability in the determination of HOMA-IR cutoff levels to define insulin resistance. The distribution of HOMA-IR varies according to the demographic characteristics of the subjects, such as age, sex, and race, making it difficult to estimate the optimal cutoff point. New indices for IR detection have been discussed. The Tg/HDL index is a useful tool for assessing glycemic control and is positively correlated with HbA1c and HOMA-IR. Therefore, Tg/HDL is thought to be a simple and inexpensive alternative to assess glycemic control in patients with prediabetes.^[29]^ In our study, a statistically significant positive weak correlation was found between uric acid and Tg/Hdl values (*R* = 0.357; *P* < .001). Some hypotheses exist about the relationship between Tg/Hdl and uric acid. Large amounts of free fatty acids (FFAs) are required for triglyceride synthesis. FFA formation by de novo synthesis of purines also accelerates uric acid production.^[30]^ Increased TG production with visceral fat accumulation is associated with higher uric acid production. It is hypothesized that TG synthesis requires NADPH, which leads to increased SUA production.^[31]^ Liu et al reported that the TG/HDL-C ratio was positively associated with the risk of hyperuricemia in the Chinese population.^[32]^ In a study conducted with 687 patients with type 2 DM, it was shown that a high serum uric acid level was associated with insulin resistance (*P* < .01), and Tg/HDL value was found to be the best marker indicating a high uric acid level (AUC:0.768).^[33]^ Babic et al showed that the Tg/HDL ratio may be a useful predictor of glycemic control in normal-weight Type 2 DM patients.^[34]^ A multivariate logistic regression analysis showed that Tg/HDL and METS-IR (the Metabolic Score for Insulin Resistance) were significantly associated with hyperuricemia.^[35]^

In our study, triglyceride levels were found to be associated with insulin resistance and uric acid levels. In a study with T2DM patients, SUA showed a significant positive correlation with TG and a significant negative correlation with HDL-C and FBS.^[36]^ In diabetes, atherogenic dyslipidemia characterized by elevated triglycerides, increased LDL cholesterol and low HDL is observed. When these are accompanied by obesity, metabolic syndrome is present. Studies have shown that first-degree relatives of diabetic patients have insulin resistance even if they are not obese.^[37]^ In a study evaluating the ratio of triacylglycerols (TAG) to HDL as an index for the diagnosis of IR, the relationship between TAG/HDL ratio and HbA1c proved to be highly significant.^[38]^ In an animal study, uric acid and triglyceride levels in the blood of animals given a high fructose diet were higher than those in the control group (*P* < .05). In this study, dapagliflozin treatment improved insulin resistance and decreased uric acid levels.^[39]^ A Peruvian study concluded that uric acid is strongly associated with metabolic syndrome in women and uric acid increases hypertriglyceridemia and IR in both sexes.^[40]^

### 4.1. Limitations

This case-control study, due to its retrospective nature, can be used to establish a correlation between variables, but not causality. Secondly, being a retrospective study, information regarding factors that could affect uric acid levels, such as high fructose, meat, alcohol consumption, body mass index, and use of antihyperuricemic agents, was not obtained, which represents a limitation of the study. Lastly, since the study was conducted in only 1 hospital, there is a potential for selection bias. Therefore, the results cannot be generalized to the entire population and should be supported by broader-based studies. In addition, the inclusion of patients with a history of hypertension, which may affect the uric acid level, is one of the limitations of this study.

## 5. Conclusion

The association between serum uric acid levels and insulin resistance has been observed. Individuals with elevated serum uric acid levels should be closely monitored for potential insulin resistance and the risk of diabetes. It would be beneficial to form a consensus on how the assessment of uric acid levels can aid preventive medicine practices, particularly when family physicians aim to prevent diabetes. To further understand the risk factors linked to variations in uric acid levels, systematic and extensive studies are essential.

## Author contributions

**Conceptualization:** Ayça Asma Sakalli.

**Data curation:** Ayça Asma Sakalli.

**Formal analysis:** H.Seda Küçükerdem.

**Investigation:** Ayça Asma Sakalli.

**Methodology:** H.Seda Küçükerdem, Olgu Aygün.

**Project administration:** Ayça Asma Sakalli.

**Supervision:** Olgu Aygün.

**Visualization:** H.Seda Küçükerdem, Olgu Aygün.

**Writing – original draft:** Ayça Asma Sakalli.

**Writing – review & editing:** H.Seda Küçükerdem, Olgu Aygün.
